# Luteolin-7-*O*-β-d-glucuronide Ameliorates Cerebral Ischemic Injury: Involvement of RIP3/MLKL Signaling Pathway

**DOI:** 10.3390/molecules29071665

**Published:** 2024-04-07

**Authors:** Xing Fan, Fang Lin, Yu Chen, Yuling Dou, Ting Li, Xinxin Jin, Jintao Song, Fang Wang

**Affiliations:** 1 School of Life Science and Biopharmaceutics, Shenyang Pharmaceutical University, Shenyang 110016, China; fanxingspu@163.com (X.F.); linfang66889@sina.com (F.L.); wangchongc@yeah.net (Y.D.); leeting321@126.com (T.L.); 2 School of Functional Food and Wine, Shenyang Pharmaceutical University, Shenyang 110016, China; chenyu2769@163.com (Y.C.); 18896656392@163.com (J.S.); 3 Department of Pharmacy, Ezhou Central Hospital, Ezhou 436000, China; 4 Experimental Teaching Center of Pharmacology, Shenyang Pharmaceutical University, Shenyang 110016, China; lingyuan-009@163.com

**Keywords:** luteolin-7-*O*-β-d-glucuronide, cerebral ischemia, oxygen–glucose deprivation, middle cerebral artery occlusion, necroptosis

## Abstract

Luteolin-7-O-β-d-glucuronide (LGU) is a major active flavonoid glycoside compound that is extracted from *Ixeris sonchifolia (Bge.)* Hance, and it is a Chinese medicinal herb mainly used for the treatment of coronary heart disease, angina pectoris, cerebral infarction, etc. In the present study, the neuroprotective effect of LGU was investigated in an oxygen glucose deprivation (OGD) model and a middle cerebral artery occlusion (MCAO) rat model. In vitro, LGU was found to effectively improve the OGD-induced decrease in neuronal viability and increase in neuronal death by a 3-(4,5-Dimethylthiazol-2-yl)-2,5-diphenyltetrazolium bromide (MTT) assay and a lactate dehydrogenase (LDH) leakage rate assay, respectively. LGU was also found to inhibit OGD-induced intracellular Ca^2+^ overload, adenosine triphosphate (ATP) depletion, and mitochondrial membrane potential (MMP) decrease. By Western blotting analysis, LGU significantly inhibited the OGD-induced increase in expressions of receptor-interacting serine/threonine-protein kinase 3 (RIP3) and mixed lineage kinase domain-like protein (MLKL). Moreover, molecular docking analysis showed that LGU might bind to RIP3 more stably and firmly than the RIP3 inhibitor GSK872. Immunofluorescence combined with confocal laser analyses disclosed that LGU inhibited the aggregation of MLKL to the nucleus. Our results suggest that LGU ameliorates OGD-induced rat primary cortical neuronal injury via the regulation of the RIP3/MLKL signaling pathway in vitro. In vivo, LGU was proven, for the first time, to protect the cerebral ischemia in a rat middle cerebral artery occlusion (MCAO) model, as shown by improved neurological deficit scores, infarction volume rate, and brain water content rate. The present study provides new insights into the therapeutic potential of LGU in cerebral ischemia.

## 1. Introduction

An acute cerebrovascular event, known as a stroke, is characterized by high morbidity, high mortality, and significant disability. It remains a leading cause of death and lifelong disability globally, primarily due to limited treatment options for ischemic brain injury [1,2]. The loss of oxygen and loss of glucose, the two crucial cellular insults that take place during cerebral ischemia, can both activate a cascade of events that disrupts cellular homeostasis and culminates in ischemic neuronal injury [3]. Currently, therapeutic approaches for cerebral ischemic stroke primarily include thrombolysis, enhancing microcirculation, restoring blood supply to ischemic areas, anticoagulation, reducing blood viscosity, neuroprotection, minimizing ischemic cerebral edema, and addressing complications [4,5]. Alongside synthetic chemicals, numerous natural products and herbal extracts have displayed efficacy in treating cerebral ischemia [6].

*Ixeris sonchifolia (Bge.)* Hance is a well-known folk medicine that is abundantly distributed throughout the northeastern part of China. It has the characteristics of invigorating blood circulation, eliminating blood stasis, and relieving pain [7,8,9]. An injectable formulation containing the raw extract of *Ixeris sonchifolia (Bge.)* Hance has been reported to exhibit various activities, including an anti-inflammatory effect, the relaxation of vascular smooth muscles, the inhibition of thrombus formation, and neuroprotective effects with relatively low side effects [8,10]. The injection preparation is mainly used for the treatment of coronary heart disease and angina pectoris, and it has also been used in the treatment of cerebral infarction for many years in China. Phytochemical investigation of this folk medicine has shown that 130 chemical constituents have been isolated from *Ixeris sonchifolia (Bge.)* Hance, including flavones, sesquiterpenes, triterpenes, phenylpropanoids, organic acids, and others [11,12,13,14].

**Figure 1:**
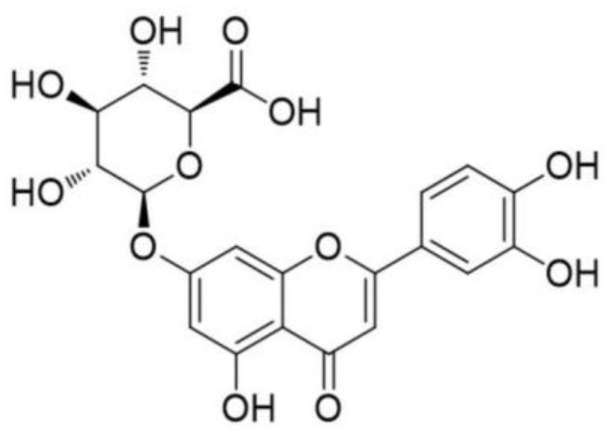
Chemical structure of Luteolin-7-β-*O*-glucuronide.

Luteolin-7-*O*-β-d-glucuronide (LGU) is a major active flavone glycoside compound extracted from *Ixeris sonchifolia (Bge.)* Hance (Figure 1). LGU has been reported to contain anti-stress, anti-depressant [15], anti-inflammatory [16,17], anti-oxidation [17,18], and antigenotoxic potency [19] characteristics, as well as leads to an inhibition of matrix metalloproteinases [20]. However, there have been no experimental studies about the anti-cerebral ischemia effect of LGU up to now and its possible mechanism has not been assessed.

**Figure 2:**
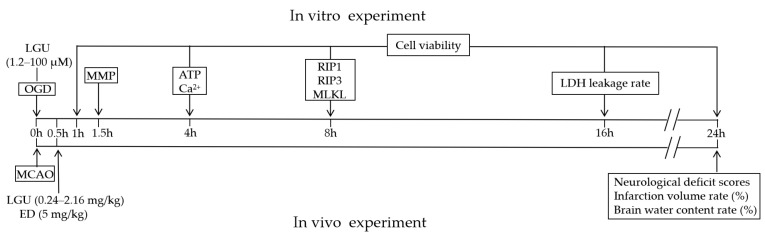
Schematic timeline of the experimental process.

During an ischemic stroke, the hypo-perfusion of neuronal tissues occurs, resulting in a localized depletion of oxygen and glucose. This leads to the failure of energy-dependent cellular processes that are crucial for cell survival [21]. In addition, it results in the failure of mitochondrial membrane integrity, further worsening the depletion of cellular energy [21]. It is well known that one of the main events occurring in ischemic brain stroke is cell death. Necroptosis is one of the modalities of cell death that accompanies ischemic brain stroke [2]. It is a regulated type of cell death mediated by receptor-interacting serine/threonine-protein kinase 1 (RIP1), RIP3, and mixed lineage kinase domain-like protein (MLKL), which results in membrane permeabilization [2,21]. The present study aims to examine whether LGU elicits a neuroprotective effect in an OGD-induced rat primary cortical neuronal injury model and an anti-cerebral ischemia effect in a middle cerebral artery occlusion (MCAO) rat model (Figure 2). This study also focused on the signaling pathway involved in necroptosis to investigate the potential mechanism.

## 2. Results

### 2.1. Protective Effect of LGU on the OGD-Induced Decrease in Cell Viability in Rat Primary Cortical Neurons

**Figure 3:**
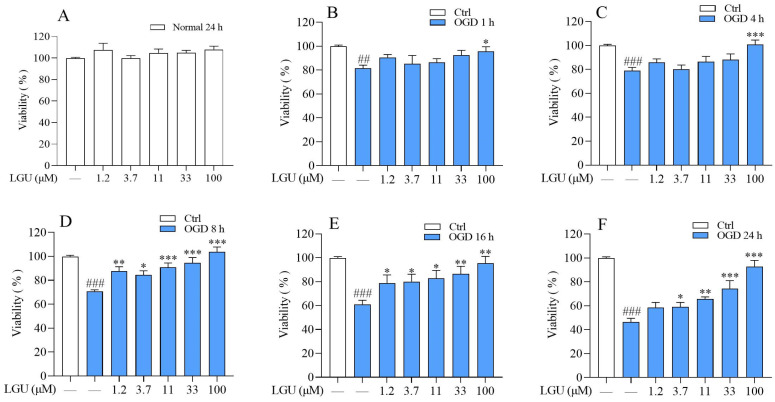
The protective effect of LGU on the OGD-induced decrease in cell viability in rat primary cortical neurons. (**A**) The effect of LGU on cell viability of primary cortical neurons (incubation: 24 h). (**B**–**F**) The protective effect of LGU on cell viability after OGD exposure (OGD 1–24 h). Data are expressed as the mean ± SEM, *n* = 3. ##: *p* < 0.01, ###: *p* < 0.001 vs. ctrl group; *: *p* < 0.05, **: *p* < 0.01, and ***: *p* < 0.001 vs. OGD group.

Compared to the control group, the cells in the OGD model group exhibited lower cell viability at 1 h, 4 h, 8 h, 16 h, and 24 h after OGD exposure (Figure 3B–F). LGU effectively attenuated the OGD-induced decrease in cell viability with different degrees (Figure 3B–F).

### 2.2. Protective Effect of LGU on OGD-Induced Neuronal Death in Rat Primary Cortical Neurons

**Figure 4:**
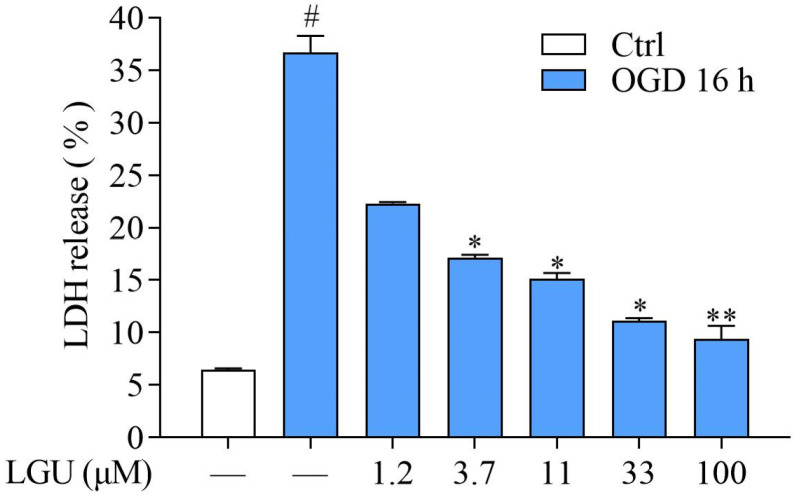
The protective effect of LGU on OGD-induced neuronal death in rat primary cortical neurons. Data are expressed as the mean ± SEM, *n* = 3. #: *p* < 0.05 vs. ctrl group, *: *p* < 0.05, and **: *p* < 0.01 vs. OGD group.

To clarify whether LGU exhibited a protective effect on OGD-induced cell death in the rat primary cortical neurons, the LDH leakage rate was detected by the LDH kit. Compared to the control group, cells in the OGD model group exhibited a higher LDH leakage rate 16 h after OGD exposure. LGU (3.7–100 μM) decreased OGD-induced LDH release (Figure 4). These results in Figure 3 and Figure 4 indicate that incubation with LGU ameliorated OGD-induced neuronal injury in rat primary cortical neurons.

### 2.3. Inhibitory Effect of LGU on OGD-Induced Intracellular Ca^2+^ Overload in Rat Primary Cortical Neurons

**Figure 5:**
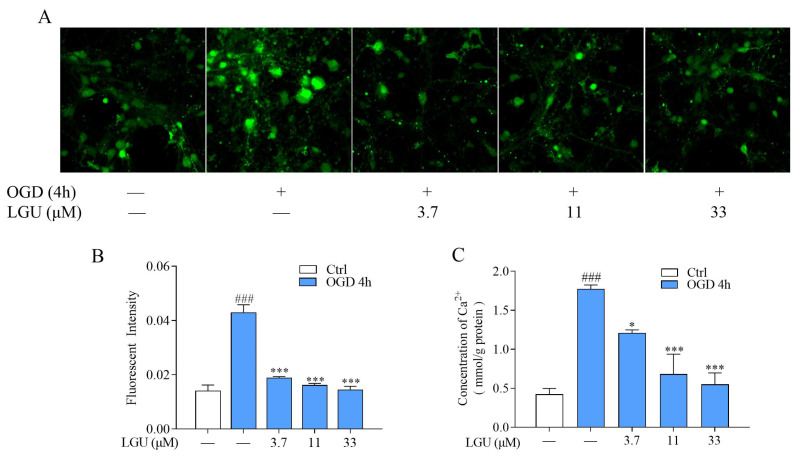
The inhibitory effect of LGU on OGD-induced intracellular calcium overload in rat primary cortical neurons. (**A**) Representative image of intracellular Ca^2+^ fluorescent intensity detected by Fluo-3/AM probe and CLSM (600×). (**B**) Quantitative analysis of LGU on the intracellular Ca^2+^ fluorescent intensity at 4 h after OGD exposure. (**C**) Quantitative analysis of the inhibitory effect of LGU on intracellular Ca^2+^ concentration at 4 h after OGD exposure detected by Ca^2+^ assay kit. Data are expressed as the mean ± SEM, *n* = 3. ###: *p* < 0.001 vs. ctrl group; *: *p* < 0.05, and ***: *p* < 0.001 vs. OGD group.

The inhibitory effect of LGU on intracellular calcium overload was investigated by using two different methods. By using a Fluo-3/AM probe and confocal laser scanning microscope, the representative images of intracellular Ca^2+^ fluorescent intensity were obtained, as shown in Figure 5A. The increase in the intracellular Ca^2+^ fluorescent intensity in rat primary cortical neurons was observed 4 h after OGD exposure; meanwhile, this effect was significantly inhibited by LGU (3.7, 11, and 33 mM) (Figure 5B). A similar result was also observed by using a Ca^2+^ assay kit, where the intracellular Ca^2+^ concentration was significantly increased; meanwhile, LGU (3.7, 11, and 33 μM) decreased the enhanced intracellular Ca^2+^ concentration in a dose-dependent manner (Figure 5C).

### 2.4. Improvement Effect of LGU on OGD-Induced ATP Depletion in Rat Primary Cortical Neurons

**Figure 6:**
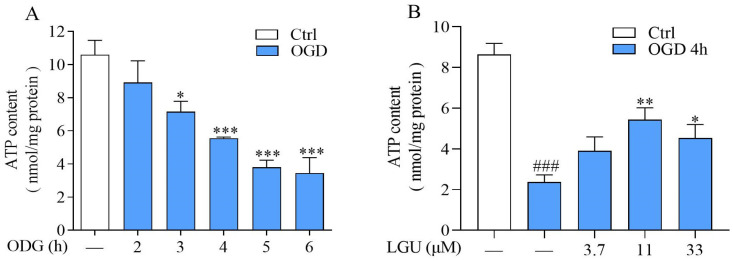
The improvement effect of LGU on OGD-induced ATP depletion in rat primary cortical neurons. (**A**) The change in ATP concentration at 2–6 h after OGD exposure. *n* = 3. *: *p* < 0.05 and ***: *p* < 0.001 vs. ctrl group. (**B**) The effect of LGU on ATP concentration 4 h after OGD exposure. *n* = 6. ###: *p* < 0.001 vs. ctrl group; *: *p* < 0.05, and **: *p* < 0.01 vs. OGD group. Data are expressed as the mean ± SEM.

To study the effect of LGU on OGD-induced ATP depletion, the intracellular ATP concentration in rat primary cortical neurons was detected by using an ATP kit. The results showed that intercellular ATP concentration in rat primary cortical neurons decreases after OGD exposure in a time-dependent manner (Figure 6A), and incubation with LGU (11 and 33 μM) significantly enhances ATP concentration at 4 h after OGD exposure (Figure 6B).

### 2.5. Improvement Effect of LGU on OGD-Induced Mitochondria Disfunction in Rat Primary Cortical Neurons

**Figure 7:**
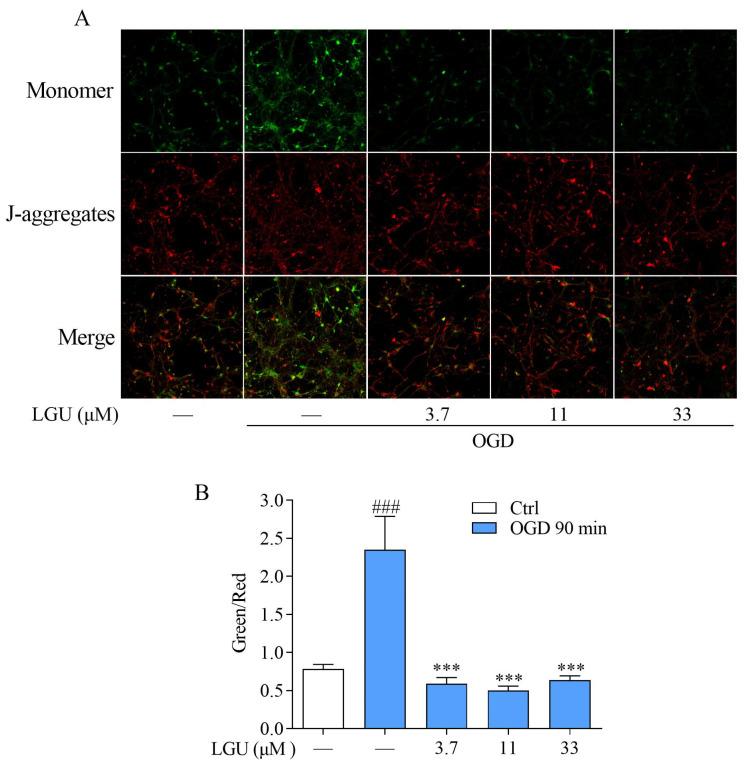
The improvement effect of LGU on OGD-induced mitochondrial damage in rat primary cortical neurons. (**A**) Representative image of the MMP in cerebral cortical neurons detected by using a JC-1 probe and CLSM (400×). (**B**) Quantitative analysis of the effect of LGU on mitochondrial damage at 90 min after OGD exposure. The change in the MMP was characterized by the change in the mean green/red fluorescence intensity ratio. Data are expressed as the mean ± SEM, *n* = 3. ###: *p* < 0.001 vs. ctrl group and ***: *p* < 0.001 vs. OGD group.

The mitochondrial membrane potential (MMP) was detected with a potentially sensitive molecular probe JC-1. When the mitochondrion was damaged, the JC-1 aggregates (red fluorescence) declined, and the accumulation of the JC-1 monomer (green fluorescence) increased. Therefore, the mean green/red fluorescence intensity ratio was negatively correlated with the MMP. The representative images of the MMP fluorescent intensity are shown in Figure 7A. As shown in Figure 7B, the neurons in the OGD group showed a decrease in the MMP, as characterized by the increased mean green/red fluorescence intensity ratio. Incubation with LGU significantly restored the MMP, and this was characterized by the decreased mean green/red fluorescence intensity ratio.

### 2.6. Improvement Effect of LGU on the OGD-Induced Activation of Necroptosis Signaling Pathway in Rat Primary Cortical Neurons

**Figure 8:**
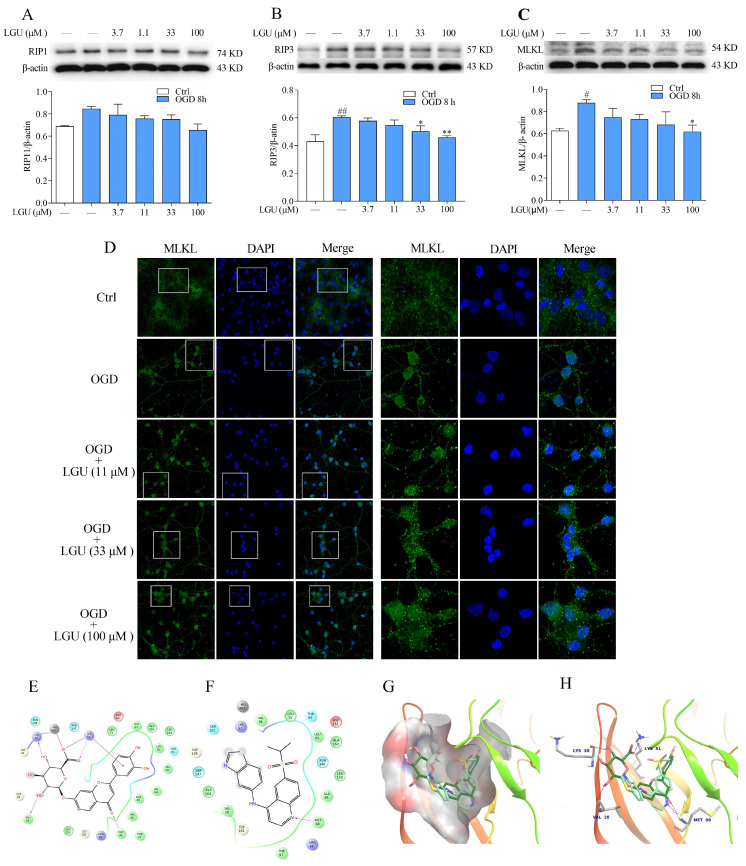
The improvement effect of LGU on the OGD-induced activation of the necroptosis signaling pathway in rat primary cortical neurons. (**A**–**C**) The expressions of RIP1 (**A**), RIP3 (**B**), and MLKL (**C**) were detected by Western blotting assay. (**D**) The representative images of expression and the nuclear translocation of MLKL (green) were detected by immunofluorescence combined with laser confocal analyses (on the left: 200×; on the right: 600×). Selected representative sections are circled with white square. DAPI (blue) was used for staining nuclei. (**E**,**F**) The 2D interaction map of LGU (**E**) and GSK872 (**F**) with the active site of RIP3. (**G**,**H**) The surface (**G**) and alignment (**H**) of GSK872 (light green) and LGU (dark green) inside the active binding site of RIP3 in the Maestro 3D. Data are expressed as the means ± SEM, *n* = 3. #: *p* < 0.05, ##: *p* < 0.01 vs. ctrl group, *: *p* < 0.05, and **: *p* < 0.01, vs. OGD group.

Western blotting assay showed that the expression of RIP1 did not exhibit a statistically significant change 8 h after OGD treatment (Figure 8A). The expression of RIP3 were significantly increased 8 h after OGD treatment compared with the control group (Figure 8B). Incubation with LGU (33 and 100 μM) significantly reduced the expression of RIP3 (Figure 8B). Compared with the control group, the expression of MLKL in the model group rats significantly increased, and the increase was significantly ameliorated by the LGU (100 μM) (Figure 8C). Moreover, immunofluorescence combined with confocal laser analyses has disclosed that MLKL has a weak fluorescence intensity (green fluorescence), and this was located extranuclearly in the control group. The green fluorescence translocated to the nucleus and formed large aggregates near the nuclear membrane at 8 h after OGD. After co-incubation with LGU, the green fluorescence was translocated to the cytoplasm (Figure 8D). From the Maestro 2D (Figure 8E,F) and 3D (Figure 8G,H), the results showed that the LGU and RIP3 inhibitor GSK872 occupied almost the same cavity of RIP3 and that it was bonded with the amino acid residues in RIP3 to form hydrogen bond at Met-98; however, LGU also formed hydrogen at Val-28, Lys-30, and Lys-51. Moreover, the binding energy of LGU (−13.720 kcal/mol) was lower than that of the RIP3 inhibitor GSK872 (−8.018 kcal/mol), thereby indicating that LGU interacted with RIP3 more stably and firmly than the RIP3 inhibitor GSK872.

### 2.7. Protective Effect of LGU on Ischemic Brain Injury in MCAO Rats

**Figure 9:**
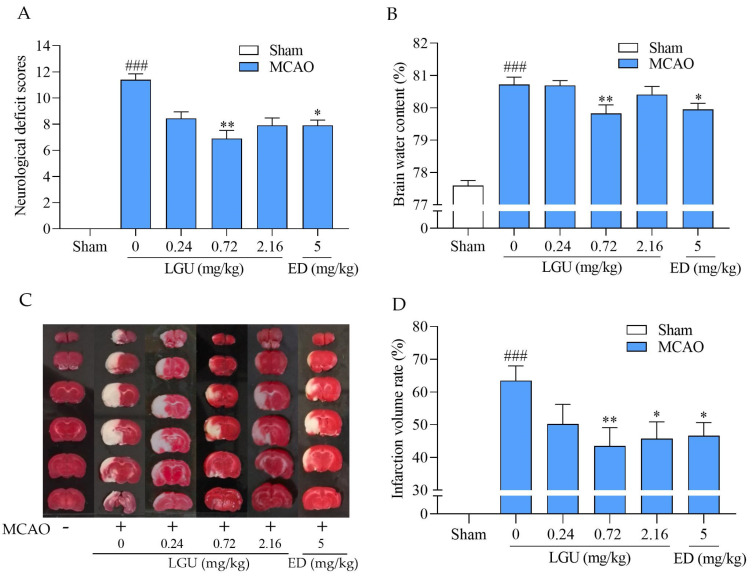
The protective effect of LGU on ischemic injury in MCAO rats. (**A**) neurological deficit scores; (**B**) brain water content rate (%); (**C**) infarction volume rate (%); and (**D**) representative images of brain slices by TTC staining. LGU (0.24, 0.72, and 2.16 mg/kg) and the positive control drug ED (5 mg/kg) were injected into the tail vein at 0.5 h after MCAO. The neurological deficit scores, infarction volume rate, and brain water content were evaluated at 24 h after MCAO. Data are expressed as the mean ± SEM. The adult male SD rats weighed 280–300 g, *n* = 8–12. ###: *p* < 0.001 vs. sham group, *: *p* < 0.05, **: *p* < 0.01 vs. MCAO group, and ED: edaravone.

As is shown in Figure 9, the neurological deficit scores, brain water content rate, and infarction volume rate were significantly increased in the MCAO rats compared to the sham group. However, the neurological deficit scores, brain water content rate, and infarction volume rate were improved by LGU at a dose of 0.72 mg/kg, and the infarction volume rate were improved by LGU at a dose of 2.16 mg/kg. The positive control drug edaravone (ED) at a dose of 5 mg/kg significantly improved three of the indicators.

## 3. Discussion

Ischemic strokes, which account for the majority of all strokes, can occur due to the temporary or permanent blockage of cerebral blood vessels [22,23]. While there have been advancements in pharmacological and mechanical thrombolysis for the treatment of ischemic stroke, the available options remain limited. Therefore, there is a significant need to develop therapeutic agents that provide neuroprotection during acute ischemic stroke, thereby safeguarding the brain from damage and enhancing functional outcomes [23]. The use of natural medicines, particularly those derived from plants, has garnered increasing interest as they possess multiple beneficial effects, thus suggesting their potential in treating cerebral ischemic stroke [24].

In the present study, we first selected 1.2, 3.7, 11, 33, and 100 μM of LGU, which did not affect the neuronal survival after 24 h of treatment, to investigate its protective effect against OGD-induced primary cortical neuron injury in vitro. During cerebral ischemia, two critical cellular insults occur, namely the deprivation of oxygen and glucose. These insults have the potential to trigger a series of events that ultimately result in neuronal death [3]. OGD is widely used as a simple and highly useful in vitro model for the elucidation of the role of the key cellular and molecular mechanisms underlying brain ischemia, which have shown similarities with the in vivo models of brain ischemia [25]. To evaluate the efficacy of LGU more comprehensively, further doses of LGU (1.2, 3.7, 11, 33, and 100 μM) and more detection time points (1 h, 4 h, 8 h, 16 h, and 24 h) were chosen, and the MTT method was used to detect the protective effect of LGU on the OGD-induced decrease in cell viability. The results showed that LGU effectively attenuates the OGD-induced decrease in cell viability with different degrees. Especially 16 h after OGD, all doses of LGU significantly improved the cell survival rate in a concentration-dependent manner. To evaluate the efficacy of LGU from different perspectives, we further chose 16 h after OGD as the representative time point to detect the LDH leakage rate to analyze the improvement effect of LGU on OGD-induced neuronal death. Our results demonstrated that LGU significantly improves OGD-induced neuronal injury through the two different methods of cell survival rate detection and cell death rate detection. However, LGU was only found to have a protective effect on OGD-induced neuronal injury in this study. Whether LGU can protect the nerve injury induced by reoxygenation after OGD needs further study.

Numerous mechanisms come into play in ischemic stroke, including mitochondrial impairment, excitotoxicity, autophagy, oxidative stress, inflammation, and damage to the blood–brain barrier [26]. As the cellular powerhouse, mitochondria play a crucial role in maintaining cellular homeostasis across various aspects of cell physiology and pathophysiology [1]. The continuous availability of ATP is essential to maintain normal neuronal functions [6]. The OGD strongly affects mitochondrial activity, thus leading to ATP depletion [27]. Many studies have indicated that a strong intracellular Ca^2+^ overload is associated with damage to neurons after ischemia. The concurrent accumulation of Ca^2+^, which reduces ATP synthesis and increases ATP usage, has been suggested as a primary cause of cell death [28]. Within minutes of an ischemic stroke, mitochondrial dysfunction emerges, leading to ATP depletion, membrane depolarization, and subsequent intracellular Ca^2+^ overload. These pathophysiological mechanisms intertwine and align with the progression of ischemic stroke [26]. These indicate that, before there was a significant decrease in the OGD-induced cell survival rate, the MMP, Ca^2+^ concentration, and ATP content change significantly, so we selected the appropriate and representative time point to detect the MMP, Ca^2+^ concentration, and ATP content in the present study. We found that LGU inhibited the intracellular Ca^2+^ overload, increased intracellular ATP concentration, and protected MMP after OGD treatment, thereby suggesting that LGU could prove to be a therapeutic candidate for the treatment of ischemic cerebral stroke.

It is well recognized that no matter what mechanism is responsible for cerebral ischemic injury, death is the destiny for most injured brain cells when subjected to ischemia. Necroptosis, which is caspase-independent cell programmed necrosis, significantly contributes to the negative events that occur with ischemic brain stroke, and its inhibition has been proven to be protective both in vitro and in vivo [21,29,30]. The RIP1-RIP3-MLKL signaling pathway is generally considered to be a marker of cell necroptosis [31]. It has been reported that energy depletion, oxidative stress, and ROS accumulation cause necroptosis [32]. When ATP production is decreased and there is not enough of it to maintain the activity and function of caspase-8 [32,33], RIP1 will interact with RIP3 to form the necrosome, which eventually leads to necroptosis [34]. Kaiser [33] reported that ischemic insults could induce an upregulation of endogenous RIP3 protein levels, which might lead to the formation of the necrosome complex and eventually necroptotic cell death. Our results showed that LGU markedly reduces the OGD-induced increased expression of RIP3. Moreover, molecular docking analysis showed that LGU might bind to RIP3 more stably and firmly than the RIP3 inhibitor GSK872. Our results demonstrate that LGU can interact with RIP3, and it can also suppress its high expression, which is induced by OGD. Previous studies have shown that when RIP3 is oligomerized, it phosphorylates further and activates the MLKL protein. Then, the activated MLKL is multimerized within the cytoplasm, giving rise to the formation of a necrosome [21]. A crucial role of the nuclear–cytoplasmic shuttling of MLKL in pro-necroptotic signaling has been unveiled recently in animal models and patients [35,36]. The association of MLKL with the cell membrane in necroptotic death is preceded by the translocation of phosphorylated MLKL to the nucleus [37]. In our present study, WB analysis showed that LGU markedly reduces the OGD-induced increased expression of MLKL. By immunofluorescence combined with confocal laser analyses, we found that the MLKL translocated to the nucleus and formed large aggregates near the nuclear membranes after OGD, and then LGU improved the translocation and aggregation. Our results suggest that LGU may inhibit the occurrence of necroptosis in primary, cultured rat cortical neurons via the regulation of the RIP3/MLKL signaling pathway.

The improvement effect of LGU on permanent focal cerebral ischemia was further studied in the MCAO rat model, and this revealed the potential therapeutic effect of LGU on cerebral ischemia in vivo. The ideal anesthetics of the experimental stroke model should meet not only potency, safety, and analgesic efficacy, but also be free of neuroprotective effects or synergistic neuroprotective effects with the test substance [38]. Compared with inhaled anesthetics that induce partial neuroprotection, chloral hydrate, the traditionally anesthetic drug, induced a higher success rate of cerebral ischemia [39]. However, due to the difference in administration method, dosage, frequency of administration, and experimental scheme, it is still controversial whether chloral hydrate has an analgesic effect [40,41,42,43,44]. Moreover, the narrow safety margin of chloral hydrate is limited to use as an anesthetic. Edaravone is an effective and well-tolerated neuroprotective agent for patients with ischemic stroke [45,46]. Therefore, edaravone was selected as the positive control drug in this study. The intravenous injection of LGU after MCAO resulted in a significant reduction in the neurological deficit scores, infarction volume rate, and brain water content rate, suggesting the potential neuroprotective effects of LGU on permanent focal cerebral ischemia. The positive control drug edaravone also significantly improved three of the indicators. Although this study revealed the therapeutic potential of LGU in permanent focal cerebral ischemia, the mechanism of LGU and the effect of LGU on other types of cerebral ischemia, such as cerebral ischemia–reperfusion injury, need to be further studied.

## 4. Materials and Methods

### 4.1. Materials

The LGU was kindly provided by Tonghua Huaxia Pharmaceutical Co., Ltd., Tonghua, China. The DMEM/F12, neurobasal medium, fetal bovine serum, B27 supplement, Trypsin-EDTA, and glucose-free DMEM were purchased from Gibco Invitrogen (Carlsbad, CA, USA). The MTT, DMSO, and Poly-L-lysine were obtained from Sigma–Aldrich (St. Louis, MO, USA). The ATP Assay Kit, Mitochondrial Membrane Potential Assay Kit (JC-1), BCA Protein Assay Kit, and RIPA lysis were obtained from Beyotime Biological (Shanghai, China). Fluo-3 AM was obtained from Dojindo Laboratories (Kumamoto, Japan). The LDH Kit and Ca^2+^ Concentration Assay Kit were purchased from Nanjing Jiancheng Bioengineering Institute (Nanjing, China). The antibodies for RIP1, RIP3, and MLKL were obtained from BD Biosciences (San Jose, CA, USA), Abcam (Cambridge, UK), and Biorbyt (Cambridge, UK), respectively. Antirabbit/mouse HRP-conjugated secondary antibodies were obtained from Zhongshan Jinqiao Biotechnology Co., Ltd. (Beijing, China). The anti-rabbit highly cross-adsorbed secondary antibody (Alexa Fluor™488) was obtained from Thermo Fisher (Beijing, China). Other general agents were available commercially.

### 4.2. Experimental Animal

Neonatal Sprague–Dawley rats, i.e., within 24 h old, and adult male Sprague–Dawley rats weighing 280–300 g (7–8 w) were purchased from Changsheng Bioscience Co., Ltd. (Benxi, China, License: SYXK (Liao) 2015-0001). The rats had access to food and water ad libitum, and they were allowed to adapt to the environment for four days prior to the experiments. The experiments were performed at a room temperature of 22–24 °C and a room humidity of 40–60%. The experimental protocol used in this study was approved by the Animal Ethics Committee of Shenyang Pharmaceutical University. The routine experiments were performed in accordance with the “Regulations on the Management of Laboratory Animals” issued by the National Science and Technology Commission. The neonatal Sprague–Dawley rats were used for the in vitro OGD experiment. The adult male Sprague–Dawley rats were used for the in vivo MCAO experiment.

### 4.3. Primary Culture of Cerebral Cortical Neurons

The primary, cultured cortical neurons were isolated and cultured, as was described previously with slight modification [47]. Briefly, after being dissected from the brain of a neonatal Sprague–Dawley rat, the cortex was treated with 0.25% trypsin for 15 min at 37 °C. The cells were collected by absorbing the supernatant and resuspended in DMEM/F12 with a 10% (*v*/*v*) fetal bovine serum, 100 U/mL of penicillin, and 100 U/mL of streptomycin. Cells were seeded onto poly-D-lysine-coated 48-well plates and were maintained in an incubator (SANYO, Osaka, Japan) in the air with 5% CO_2_ and at 37 °C. After 4 h, the culture medium was changed to a neurobasal medium, which was supplemented with 2% B27, 100 U/mL of penicillin, and 100 U/mL of streptomycin. The medium replacement was performed every 72 h. The neurons were maintained for 6–7 days in a primary culture until used for the following experiment.

### 4.4. Oxygen–Glucose Deprivation (OGD) and Drug Treatment

The procedures for OGD were performed as was described previously with slight modification [48]. The primary neurons in the control group were cultured with glucose-containing media in a 37 °C/5% CO_2_ incubator. The LGU was dissolved in DMSO and diluted with glucose-free DMEM saline to make the concentration of the DMSO one-thousandth of the final volume. Primary neurons in the model group and LGU groups were cultured with glucose-free DMEM containing 0.1% DMSO and different doses of LGU, respectively. Meanwhile, the neurons in the model group and LGU groups were placed in a hypoxic incubator (Stemcell Technologies, Vancouver, BC, Canada), which were filled with mixed gas containing 95% N_2_ 5% and CO_2_ at 37 °C to induce OGD injury.

### 4.5. Cell Viability Assay

After different times of OGD exposure, the cultures were incubated with an MTT solution (0.25 mg/mL) for 4 h at 37 °C. Then, the medium was discarded and DMSO was added to solubilize the reaction product formazan with shaking for 15 min. The optical density (OD) was read at 570 nm using a Synergy HT microplate reader (GENE, Oceanside, CA, USA). Each experiment was performed using three replicated wells for each group. The percentage of cell viability (CV %) was calculated by the following formula:

$$cv(\%)=\frac{OD(Sample)}{OD(Control)}\times 100\%.$$

### 4.6. Lactate Dehydrogenase (LDH) Assay

To confirm neuron death, the LDH activity in the medium at 16 h after OGD was determined according to the protocols of an LDH kit. Briefly, after treatment, the medium was collected, according to the manufacturer’s protocol, to measure the LDH activity. Absorbance at 450 nm was measured in a microplate reader (Synergy HT, BioTek, Phoenix, AZ, USA). The LDH leakage was expressed as the percentage (%) of the total LDH activity (LDH in the medium + LDH in the cell) [49].

$$LDH\;leakage\;(\%)=\frac{LDH\;in\;the\;medium}{LDH\;in\;the\;medium+LDH\;in\;the\;cell}\times 100\%$$

### 4.7. Detection of Intracellular Calcium ([Ca^2+^]i)

The fluorescence images (600× magnification) were captured using a confocal laser scanning microscope (CLSM) (Nikon, Tokyo, Japan). The [Ca^2+^]i was measured at 4 h after OGD with the fluorescent dye Fluo 3-AM according to the manufacturer’s protocol. The intracellular Ca^2+^ levels were also determined by the Ca^2+^ concentration assay kit according to the manufacturer’s protocol.

### 4.8. Detection of Intracellular ATP Level

The intracellular ATP levels were determined using an ATP bioluminescence assay kit according to the manufacturer’s protocol.

### 4.9. Measurement of the Mitochondrial Membrane Potential (MMP)

The MMP was assessed using the cell-permeable fluorescent dye, the JC-1 probe. JC-1 exists as a green, fluorescent monomer at a lower MMP, while JC-1 forms red fluorescent aggregates at a higher MMP. It can, therefore, be used as a sensitive measure of changes in the MMP [50]. The MMP of the primary cortical neuron cultures was measured at 90 after OGD by the JC-1 mitochondrial membrane potential assay kit according to the manufacturer’s protocol. The fluorescence of JC-1 in three random fields (400× magnification) in each group sample was measured by using a CLSM (Nikon, Japan), and the JC-1 fluorescence intensity was analyzed using Image-Pro Plus 6.0 software. The ratio of green fluorescence density/red fluorescence density for each sample was used as the degree of the MMP in this sample.

### 4.10. Western Blot Detection

Cellular proteins were harvested and homogenized in an RIPA lysis buffer and the total proteins were measured by a BCA kit. Separate gels of different concentrations (8%, 10%, and 12%) and 5% concentrated gels were formulated according to different proteins. The protein samples were loaded onto SDS-polyacrylamide gels and electrophoresis was performed. The proteins were transferred onto nitrocellulose membranes. The filters were blocked with 5% dry skim milk for 1 h, then the proteins were incubated with primary antibodies overnight at 4 °C, including anti β-actin (Santa Cruze, Santa Cruz, CA, USA, sc-47778, 1:1000), RIP1 (BD Biosciences, 610458, 1:1000), RIP3 (Abcam, ab62344, 1:1000), and MLKL (Biorbyt, orb32399, 1:1000) antibodies. β-actin was used as the internal reference to ensure equal loadings. After washing with TBST buffer, the membranes were incubated with HRP-conjugated secondary antibodies (HZB2305/HZB2301, 1:10,000) for 1 h at room temperature. Finally, the membranes were detected utilizing the ECL reagent and the gray intensity was measured by Image J 1.4 software (NIH, Bethesda, MD, USA).

### 4.11. Immunofluorescence Staining

The rat primary neurons were washed with PBS, fixed with 4% paraformaldehyde in PBS for 20 min at room temperature, permeabilized with 0.1% Triton X-100, and then blocked with 5% normal goat serum for 1 h at room temperature. The antibody to MLKL (Biorbyt, orb32399, 1:1000) was applied overnight at 4 °C, followed by 1 h of incubation at 37 °C with fluorescein isothiocyanate (FITC)-conjugated secondary antibodies (A-11034, 1:10,000). After being washed with PBS 3 times, the nuclei of the neurons were stained with DAPI for 10 min. The fluorescence images (400× magnification) were captured using a Nikon CLSM.

### 4.12. Molecular Docking

The experimental crystal structure of the receptor-interacting serine/threonine-protein kinase 3 (RIP3) was downloaded from the RCSB Protein Data Bank (PDB codes: 4M69) (http://www.rcsb.org/, accessed on 1 May 2018). The quaternary structure of the protein with a PDB number of 4M69 contained two chains, A and B, of which the A chain was RIP3 and the uniport sequence number was Q9QZL0. All water molecules were removed from the structures. The A chain of protein 4M69 was prepared by using the Protein Preparation Wizard module in Schrödinger version 2014, which included assigning bond orders, adding hydrogens, creating zero-order bonds to metals, and creating disulfide bonds. Grids were generated by the Glide module after determining the position of cocrystal ANP as the active site. The RIP3 inhibitor GSK872 and LGU were optimized with an OPLS-2005 force field using the Ligprep module in Schrödinger version 2014, and this was then imported into the A chain of 4M69 protein grids. The optimized GSK872 and LGU were docked into the A chain of the 4M69 protein by glide module, and the interaction between protein and conformation was then outputted.

### 4.13. MCAO Model

Adult male Sprague–Dawley rats weighing 280–300 g were randomly divided into the following six groups: the Sham group, MCAO group, LGU (0.24, 0.72, and 2.16 mg/kg) treatment groups, and the positive control drug edaravone (ED, 5 mg/kg) group. The LGU and ED groups were treated through tail intravenous injection 0.5 h after MCAO. The MCAO model was performed as was described previously with some modifications [51]. The adult male Sprague–Dawley rats weighing 280–300 g were fasted 12 h before the operation. All rats were anesthetized with chloral hydrate solution (350 mg/kg, i.p.) and then operated upon. A 0.5 cm midline right side incision was made on the neck to search and expose the external carotid artery (ECA), internal carotid artery (ICA), and common carotid artery (CCA). The proximal end of ECA and CCA were tied with sutures. The distal end of the CCA was threaded through a suture for standby, and the ICA was temporarily clipped with a mini arterial clamp. A V-shaped incision cut was made on the ECA 5 mm from where the bifurcations of the ECA, ICA, and CCA were performed, then a 5-0 nylon suture (diameter, 0.26 mm) was inserted via the ECA into the ICA until its tip reached the origin of the middle cerebral artery (MCA) to occlude the MCA. The 5-0 nylon suture was fastened by tightening the suture around the distal CCA and the neck incision was closed. The sham-operated rats received all surgical procedures but without the 5-0 nylon suture inserted.

LGU was dissolved in DMSO and diluted with saline to make the concentration of DMSO one-thousandth of the final volume. After 0.5 h of MCAO, the rats in the LGU groups and the positive drug group were immediately injected with LGU (0.24 mg/kg, 0.72 mg/kg, and 2.16 mg/kg) and the positive control drug edaravone (5 mg/kg) in the tail vein, respectively. The model group and sham operation group were injected with the same vehicle. After 24 h of MCAO, the neurological deficit scores, infarction volume rate, and brain water content were evaluated.

### 4.14. Modified Neurological Severity Score (mNSS)

Assessment of the neurological deficits was performed on rats at 24 h after MCAO by using the mNSS [52]. The mNSS is a composite of motor, sensory, reflex, and balance tests. The neurological function was graded on a scale of 0 to 18 (normal score 0; maximal deficit score 18). The higher the score, the more severe the injury.

### 4.15. 2,3,5-Triphenyl-Tetrazolium (TTC) Staining

TTC staining was used to measure the infarct volumes. The TTC staining was performed as was described previously with slight modifications [53]. Briefly, the brains were quickly taken out after MCAO and then cut into five 2 mm-thick coronal slices using the brain matrix. The fresh slices were incubated away from light in a 1% TTC solution for 0.5 h at 37 °C and then transferred into a 4% formaldehyde solution for fixation to visualize the infarctions. The normal brain tissue was stained uniformly red while the infarction region was stained white. The brain slices were photographed with a digital camera, and the sizes of the infarct area were assessed by Image J software. The results were expressed as the volume ratio of the cerebral infarction:

$$Infarct\;volume\;ratio\;(\%)=\frac{the\;infarction\;volume}{the\;ipsilateral\;hemisphere\;volume}\times 100\%.$$

### 4.16. Brain Water Content

Cerebral edema was determined after MCAO via measuring brain water content by means of the standard wet/dry weight method [54]. The brains were removed quickly and weighed to obtain the wet weight, and they were then placed in an oven to obtain their dry weight. The brain water content percentage was calculated according to the following formula:

$$Brain\;water\;content\;(\%)=\frac{Wet\;weight-Dry\;weight}{Wet\;weight}\times 100\%.$$

### 4.17. Statistical Analysis

Data were expressed as the mean ± S.E.M. Statistical analyses were performed using IBM SPSS (Version 27.0, Armonk, NY, USA). Differences in the parameters were performed by using analysis of variance (ANOVA) followed by the LSD or Dunnett test. For the non-parametric statistics test, the Kruskal–Wallis H test was performed. Statistical significance was set at *p* < 0.05.

## 5. Conclusions

In the present study, for the first time, we presented evidence that LGU has a protective effect against OGD-induced rat primary cortical neuronal injury, and its protective effect is associated with the inhibition of intracellular Ca^2+^ overload, an improvement effect in ATP depletion, and recovery of the MMP. Moreover, LGU was found to also exert a protective effect via regulation of the RIP3/MLKL signaling pathway. We confirmed, for the first time, the protective effect of LGU on cerebral ischemia in a rat MCAO model, as shown by the improved neurological deficit scores, infarction volume rate, and brain water content rate. Although further studies are needed to elucidate the deep mechanism of the anti-necroptotic effect of LGU, this study provides new insights into the therapeutic potential of LGU in cerebral ischemia.

## Data Availability

The data that support the findings of this study are available from the corresponding authors upon reasonable request.
